# Supplementary material for: Transcriptomic Analysis of Testicular Gene Expression in a Dog Model of Experimentally Induced Cryptorchidism
Source: Cells. 2022 Aug 10;11(16):2476. doi: 10.3390/cells11162476 (PMC9406621; doi:10.3390/cells11162476)
Supplement: Supplementary file 1 [file cells-11-02476-s001.zip › cells-1862560-supplementary.pdf]

A

Sample id	read type	# of processed reads	# of mapped reads	# of multiple mapped reads	overall read mapping ratio
CT-1	1	34,378,549	32,603,787 (94.8%)	2,548,209 (7.8%)	94.5%
CT-1	2	34,378,549	32,370,223 (94.2%)	2,529,419 (7.8%)	
CT-2	1	42,126,521	40,205,769 (95.4%)	1,578,223 (3.9%)	95.1%
CT-2	2	42,126,521	39,929,959 (94.8%)	1,563,956 (3.9%)	
CT-3	1	29,101,232	26,666,130 (91.6%)	1,313,170 (4.9%)	91.1%
CT-3	2	29,101,232	26,374,393 (90.6%)	1,295,528 (4.9%)	
WT-1	1	43,841,776	41,646,485 (95.0%)	1,339,185 (3.2%)	94.6%
WT-1	2	43,841,776	41,340,711 (94.3%)	1,324,487 (3.2%)	
WT-2	1	39,671,527	37,669,285 (95.0%)	1,161,932 (3.1%)	94.7%
WT-2	2	39,671,527	37,432,898 (94.4%)	1,151,360 (3.1%)	
WT-3	1	29,842,605	27,395,917 (91.8%)	1,063,193 (3.9%)	91.3%
WT-3	2	29,842,605	27,111,049 (90.8%)	1,048,334 (3.9%)	

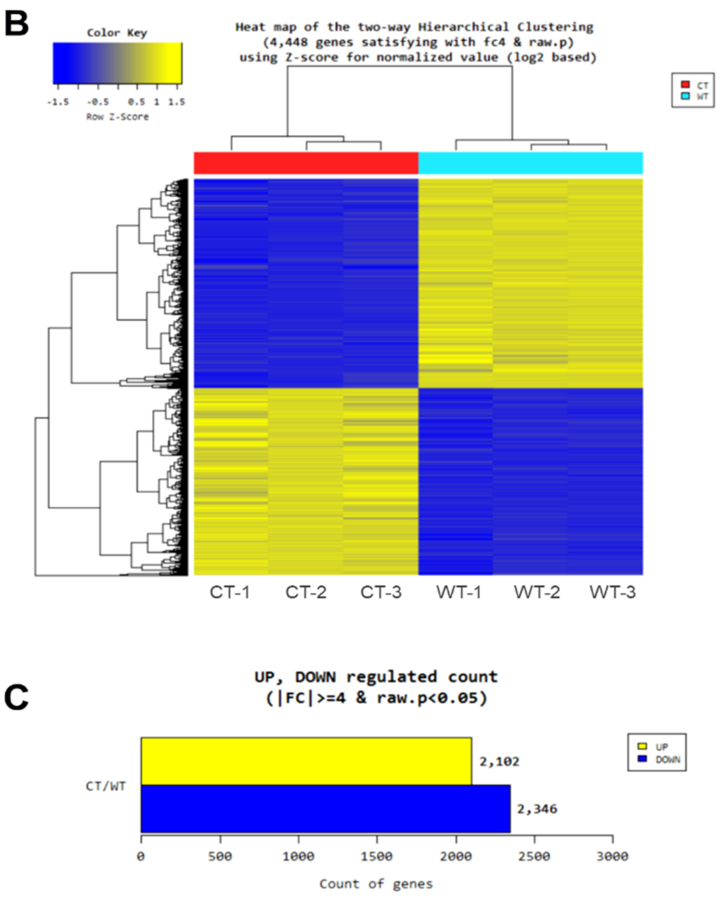

Figure S1 Statistics of RNA sequencing. (**A**) Summary of the mapping results for RNA-seq. (**B**) Heatmap of 4,448 DEGs generated by using the hierarchical clustering method. The color represents the different expression levels of genes between WT and CT. (**C**) The number of up-regulated or down-regulated genes.
